# Prevalence and patterns of Y chromosome microdeletion in infertile men with azoospermia and oligzoospermia in Northeast China

**Published:** 2014-06

**Authors:** Fadlalla Elfateh, Dai Rulin, Yun Xin, Li Linlin, Zhu Haibo, Rui-Zhi Liu

**Affiliations:** *Center for Reproductive Medicine, First Bethune Hospital, Jilin University, Changchun, China.*

**Keywords:** *Azoospermia*, *Oligozoospermia*, *Y chromosome microdeletions*, *PCR*

## Abstract

**Background:** In some cases infertile men showed small deletions of specific genes in the Y chromosome. It had been confirmed, these deleted genes are greatly associated with spermatogenic failure. However, the frequency and the patterns of such microdeletions among infertile men are not clearly clarified.

**Objective:** We sought to determine the frequency and the patterns of Y chromosome microdeletions in azoospermic and oligozoospermic infertile men in Northeast China, and try to optimize the selection of sequence tagged sites (STSs) of AZF microdeletions in multiplex polymerase chain reaction (PCR).

**Materials and Methods: **720 azoospermic and 330 oligozoospermic infertile men, from Northeast China were included in this retrospective study during May 2008 to November 2012. Semen analysis was performed according to the World Health Organization guidelines. Y chromosome microdeletions were detected by polymerase chain reaction assays. G-banding method was used for chromosome Karyotype analysis. Chi-square tests were used to compare patterns of Y chromosome microdeletions in azoospermic and oligozoospermic patients.

**Results: **Of 1050 infertile men, 12.95% cases had shown Y chromosome microdeletions, and 19.43% of cases showed abnormal chromosomal karyotype. Deletions in AZFc region was the most frequent 75.00%, followed by deletions in AZFb region 13.33%, AZFbc region 09.62%, and AZFa region 2.22%. All oligozoospermic patients showed presence of sY84, sY86, sY127, and sY134. Deletion of sY127 (p=0.0101) and sY157 (p=0.0043) showed significant difference between azoospermic group and oligozoospermic group.

**Conclusion:** Deletions of sY127 may relate to azoospermia while sY84, sY86, sY127 can be ignored in AZF screening for oligozoospermic patients.

## Introduction

It has been estimated that 10-15% of couples worldwide suffer from infertility, and male factor can be identified in about half of these cases (1). Genetic factors contribute about 10-15% of male infertility (2). Among these factors, Y chromosome microdeletions are the second most frequent genetic cause of male infertility after Klinefelter's syndrome (3). The sex determining Y chromosome is the smallest human chromosome consists of a short (Yp) and a long (Yq) arm. The majority of the microdeletions which cause azoospermia or oligozoospermia occur in the non-overlapping regions of the long arm, these microdeletions (compared with other structural chromosomal abnormalities) are too small to be detected by karyotyping. They can be identified using polymerase chain reaction (PCR). 

The first hypothesis defined the correlation between Y chromosome deletions and male infertility was reported by Tiepolo and Zuffardi in 1976 (4). Investigation of these deletions has revealed that there are three non-overlapping Y-chromosome regions important for normal spermatogenesis. These regions are AZFa, AZFb, and AZFc from proximal to distal Yq arm (5). Furthermore, a fourth region (AZFd) has been discovered later (6). Recently Y chromosome microdeletions are recognized as the most important genetic etiology of male infertility throughout the world in addition to close relation with recurrent abortion (7). 

The incidence of Y chromosomes microdeletions was described previously, most of these previous studies described the prevalence of microdeletion involving the regions and the common patterns (AZFc, AZFbc AZFb, AZFa, and AZFabc) but no one had investigated the incidence of deletion involving the sequence tagged sites STSs (sY84, sY86, sY127, sY134, sY143, sY157, sY255, sY254 and sY152) individually. 

This study aimed to determine the frequency and the patterns of Y chromosome microdeletions in infertile men with azoospermia and oligozoospermia in Northeast China, and try to reveal significant STSs among azoospermic, and oligozoospermic groups. We used multiplex PCR, to detect nine sequence tagged sites STSs (sY84, sY86, sY127, sY134, sY143, sY157, sY255, sY254, and sY152).

## Materials and methods

**Patients**

A retrospective study was performed to 1050 infertile men aged between 20 and 44 years old from Northeast China who attend the First, Hospital of Jilin University, were included in this study during May 2008 to November 2012. 720 suffering from azoospermia, and 330 from oligozoospermia. Patients with microdeletions associated with abnormal karyotype or obstructive azoospermia were excluded from this study. Patient’s medical histories, reproductive problems, and physical examination were recorded. Semen samples were obtained in the period of 2-7 days after ejaculatory abstinence, and semen analysis was performed according to the World Health Organization guidelines (8). Peripheral blood samples were obtained and stored for cytogenetic detection. Oligozoospermia was diagnosed with a sperm count <20×10^6^ /mL. And another 100 healthy fertile men were included as controls; every man in the control group had fathered at least one child. 

This study was approved by the Reproductive Medicine Ethics Committee of First Hospital of Jilin University, and all patients signed informed consents of this study before semen analysis.

**DNA extraction and** **PCR** **analysis**

Peripheral blood samples were obtained from all patients and the genomic DNA was isolated using commercially-available blood DNA extraction kits (Beijing Tiangen Biotech Co., Ltd, China). Control DNA samples obtained from unrelated normal males with proven fertility and from normal females were used as positive and negative controls respectively. A sample containing all reaction components and water in place of the DNA template was used as the PCR blank control. 

Based on the recommendations of the European Academy of Andrology (EAA) and the European Molecular Genetics Quality Network (EMQN), the samples were tested for classical Y chromosome microdeletions using the following sequence-tagged sites (STSs) proposed by Simoni *et al*: sY84, sY86, sY127, sY134, sY143, sY152, sY254, sY255, and sY157 (9). The detections of sY14 (SRY) and ZFX/ZFY were employed as internal controls. Multiplex PCR was carried out in a total volume of 30 μL using a Veriti 96-well PCR thermal cycler (Applied Biosystems, USA). The results were considered positive when a clear amplification product of the expected site was obtained. 

**Karyotype analysis**

After culturing peripheral blood samples for 72h, lymphocyte chromosome spreads were prepared using routine methods; Karyotypes were described according to the International System for Chromosome Nomenclature (ISCN), and analyzed by G-banding (10). For each individual, a minimum of 20 metaphase cells were counted and at least five cells were analyzed. 

**Statistical analysis**

Chi-square tests were used to compare patterns of Y chromosome microdeletions in azoospermic and oligozoospermic patients. Differences were considered to be statistically significant when p<0.05.

## Results

Out of the studied 1050 infertile men, there were 204 patients had shown abnormal karyotype 19.43% (204/1050), beside 136 patients had shown Y chromosome microdeletions 12.95% (136/1050) (Table I). Occurrence of microdeletions was found at a rate of 14.03% (101/720), and 10.60% (35/330) in the azoospermic and oligozoospermic men respectively. Among these deletions, AZFc region was the most frequent 75.00 %, followed by AZFb 13.33%,AZFbc 09.62%, and AZFa 2.22%%. (Table II). In the AZFa region, deletions of sY84 and sY86 were at a rate of 00.27% (1/371), and 00.54% (2/371) respectively. Microdeletions in AZFb region had an estimation of about 4.31% (16/371), 01.61% (6/371), and 02.15% (8/371) for sY127, sY134, and sY143 respectively. In AZFc region, deletions of sY152, sY254, sY255, and sY157 estimated about 22.64% (84/371), 26.68% (99/371), 26.68% (99/371), and 15.09% (56/371) respectively (Table III).

Azoospermia: For the azoospermic patients the results showed that microdeletions were detected in 101 azoospermic patients 14.03% (101/720). The most common deleted region was AZFc region 67.32% (of deletions involving azoospermic group 68/101) followed by AZFb region 16.83% (17/101), AZFbc 12.87% (13/101), and AZFa 2.97% (3/101) (Table IV). The distribution of micodeletions as the following: AZFa region estimated at about 00.4%% (1/250) and 00.8% (2/250) for sY84 and sY86 respectively. 

In AZFb region deletion of sY127, sY134 and sY143 estimated about 6.4% (16/250), 02.4% (6/250), and 02.8% (7/250) respectively. In AZFc region, deletions of sY152, sY254, sY255, and sY157, estimated about 22.4% (56/250), 26.80% (67/250), 26.80% (67/250), and 11.20% (28/250) respectively (Table V). All patients who showed absence of sY127 were diagnosed as azoospermic patients (Table VI). Microdeletions involving one sequence tagged site appeared in five cases, these were sY84 (two cases), sY86 (one case), sY127 (one case) among azoospermic group, and sY143 (one case) from oligozoospermic group (Table VI). 

Deletion of sY127 (p=0.0101) and sY157 (p=0.0043) showed significant difference between azoospermic group and oligozoospermic group. No significant difference was noted in the deletions involving the other sequence tagged sites (Table VII). Oligozoospermia: Among oligozoospermic patients, microdeletions were found in 35 patients 10.60% (35/330). The most common microdeletions were detected in AZFc region 97.14% (34/35) (Table VIII). Deletions of sY152, sY254, sY255, and sY157 estimated about 23.14% (28/121), 28.82% (32/121), 28.82% (32/121), and 23.14% (28/121) (of deletions involving oligozoospermic group) respectively (Table IX). No deletion was detected in AZFa region. For AZFb region, only one patient showed deletion in sY143 (1/121, 00.82%). All oligozoospermic patients showed presence of sY84, sY86, sY127 and sY134 (Table IX).

**Table I:** 1050 infertile patient's chromosome karyotype

**Types**	**Number (N)**	**Proportion (%)**
Klinefelter syndrome	139	13.24% (139/1050)
Polymorphic	36	3.43% (36/1050)
Translocation	15	1.43% (15/1050)
Inversion	4	0.38% (4/1050)
Sex reversal	3	0.29% (3/1050)
47,XYY	1	0.09% (1/1050)
Marker Chromosome	1	0.09% (1/1050)
Others	5	0.48% (5/1050)
Total abnormal	204	19.43% (204/1050)
Normal karyotype	846	80.57% (846/1050)

**Table II:** Microdeletions of 1050 azoospermic and oligozoospermic patients: Frequency and percentage

**AZF region deletion**	**Frequency**	**% (all deletions)**
AZFa	3	02.22%
AZFb	18	13.33%
AZFc	102	75.00%
AZFbc	13	09.62%
AZFabc	0	0.00%

% out of all deletions (n=136).

**Table III:** Sequence tagged sites STSs Microdeletions of 136 microdeletion patients: Frequency and percentage (Total 371 STSs

**STS Deletion**	**Frequency**	**% (** **371 STSs** **)**
sY84	1	00.27%
sY86	2	00.54%
sY127	16	4.31%
sY134	6	01.61%
sY143	8	02.15%
sY152	84	22.64%
sY157	56	15.09%
sY254	99	26.68%
sY255	99	26.68%

% out of all deletions (n=371).

**Table IV:** Microdeletions of 720 azoospermic patients: Frequency and percentage

**AZF region delation**	**Frequency**	**% (deletions among azoospermia)**
AZFa	3	2.97%
AZFb	17	16.83%
AZFc	68	67.32 %
AZFbc	13	12.87%
AZFabc	0	00.00%

% out of deletions among azoospermic group (n=101).

**Table V:** Sequence tagged sites STSs microdeletions of 101 azoospermic patients: Frequency and percentage (Total 250 STSs)

**STS Deletion**	**Frequency**	**% (deletions among azoospermic group)**
sY84	1	00.4%
sY86	2	00.8%
sY127	16	6.4%
sY134	6	02.4%
sY143	7	02.8%
sY152	56	22.4%
sY157	28	11.20%
sY254	67	26.80%
sY255	67	26.80%

% out of deletion among f azoospermic group (n=250).

**Table VI:** Patterns of Y chromosome microdeletions involving sY127 and semen status

**SN**	**sY86**	**sY84**	**sY127**	**sY134**	**sY143**	**sY157**	**sY254**	**sY255**	**sY152**	**Diagnosis**
1	0	0	del	0	0	del	del	del	del	azoospermia
2	0	0	del	0	0	del	del	del	del	azoospermia
3	0	0	del	del	del	0	0	0	0	azoospermia
4	0	0	del	0	0	del	del	del	del	azoospermia
5	0	0	del	0	0	del	del	del	del	azoospermia
6	0	0	del	0	0	del	del	del	del	azoospermia
7	0	0	del	del	0	0	del	del	del	azoospermia
8	0	0	del	0	0	del	del	del	del	azoospermia
9	0	0	del	0	0	del	del	del	del	azoospermia
10	0	0	del	0	0	0	0	0	0	azoospermia
11	0	0	del	0	del	0	0	0	0	azoospermia
12	0	0	del	0	0	0	del	del	del	azoospermia
13	0	0	del	del	0	del	del	del	del	azoospermia
14	0	0	del	del	del	0	0	0	0	azoospermia
15	0	0	del	del	del	0	del	del	del	azoospermia
16	0	0	del	del	del	0	0	0	0	azoospermia

SN= serial number.

0: sequence-tagged site marker present.

Del: sequence-tagged site marker absent.

**Table VII:** Distribution of Y chromosome microdeletions: comparison between azoospermic and oligozoospermic groups

**Deletion**	**Frequency in azoospermia**	**Frequency in oligospermia**	**p-value**
sY84	1	0	0.7104 ^NS^
sY86	2	0	0.8178 ^NS^
sY127	16	0	0.0101 ^S^
sY134	6	0	0.2009 ^NS^
sY143	7	1	0.3015 ^NS^
sY152	56	28	0.9781 ^NS^
sY157	28	28	0.0043 ^S^
sY254	67	32	0.9578 ^NS^
sY255	67	32	0.9578 ^NS^
Total	250	121	

Chi-square tests. Differences were considered to be statistically significant when p<0.05.

NS= Non significant

S= Significant

**Table VIII:** Microdeletions of 330 oligozoospermic patients: Frequency and percentage

**AZF region deletion**	**Frequency**	**% (deletions among oligozoospermia)**
AZFa	0	0%
AZFb	1	2.85%
AZFc	34	97.14%
AZFbc	0	00%
AZFabc	0	00%

% out of deletions among oligozoospermic group (n=35).

**Table IX:** Sequence tagged sitesoligozoospermia STSs microdeletions of 35 oligozoospermia patients: Frequency and percentage (Tota 121 STSs)

**STS Deletion**	**Frequency**	**% (deletions among oligozoospermia** ** STSs** **)**
sY84	0	00.00%
sY86	0	00.00%
sY127	0	00.00%
sY134	0	00.00%
sY143	1	00.82%
sY152	28	23.14%
sY157	28	23.14%
sY254	32	26.45%
sY255	32	26.45%

% out of deletions among oligozoospermic group (n=121).

## Discussion

This study dealt with the frequency and the patterns of Y chromosome microdeletions in infertile men in Northeast China. The results have shown that microdeletions were detected among 136 male patients out of 1050 infertile men, with a prevalence of 12.95% (136/1050). Compared with previous studies this findings revealed that, microdeletions are more prevalent among Northeast Chinese population as compared with Korean population where a percentage of 7.7% has been reported (11). The prevalence is similarly higher among Northeastern China population when compared with findings in Brazilian (7.5%) and Serbian population (5.4%) (3, 12). 

But seems to be lower than that reported for Iranian population (12%), and Southern Chinese population (12%) (13, 14). None-the-less, the presently observed prevalence is similar to those described previously in other Chinese population (11.3%, 10.9%, and 11.5%) (15-17). The present study results demonstrated that, microdeletions of AZFc region were the most frequent 75.00%, followed by AZFb 13.33%,AZFbc 09.62%, and AZFa 2.22%. These findings agree with a previous Chinese study, which concluded that, deletions of AZFc region are the most frequent 72.7%, followed by AZFbc 13.6%, AZFabc 6.1%, AZFb 4.5% and AZFa 3.0% (15). 

Our findings is partially similar with a relevant recent Korean study that revealed a high frequency of microdeletions in the AZFc region (87.1%) as compared to deletions in AZFbc 24.7% and AZFabc 8.9% (11). Moreover, in 2006 Song *et al* studied 21 cases of microdeletions among 143 infertile patients, and found that, deletions of AZFc region were at a percentage of 76.2%, of the AZFb region were at 9.5% and those of AZFa region at 4.8% (18). Furthermore, another study by Ristanovic *et al* reported the incidence of Y chromosome microdeletions as the following: deletions involving AZFc region estimated at about 75%, AZFa 8% and AZFbc 17% (9). 

In 2009 Zhou *et al* reported that, deletion of the AZFc region was the most common deletions 63.8%, followed by AZFbc 19.0%, AZFabc 10.3% and AZFb or AZFa 3.4% (19). The present study concluded that, deletion of sY255 and sY254 is the most common pattern of AZF microdeletions. Hence, this conclusion is in accordance with the findings of Wu *et al*, who had reported that, deletions of sY255 and sY254 among Chinese population is the most common pattern of AZF microdeletions (20). In comparison between azoospermic men and oligozoospermic men, previous studies found that, the prevalence of microdeletions was higher in azoospermic than in oligozoospermic men as reported by Shi *et al* (9.16% and 5.56% respectively), and Zhu *et al *(14% and 8.2% respectively) (17, 21). In our study, however, the incidence was found to be at a similar rate in the azoospermic group (101/720, 14.03%) and in the oligozoospermic group (35/330, 10.60%).

This study has revealed presence of sY84, sY86, sY127and sY134 in all oligozoospermic patients. Moreover, the present study also showed that, absence of sY84, sY86, and sY127 alone or along with other sequence tagged sites results in azoospermia. This finding is in agreement with the report of Hopps *et al* who noted that, men with microdeletion of the AZFa or AZFb regions of the Y chromosome have very poor amount of sperm, whereas the majority of male with AZFc deletion have sperms within the semen or testes (22). The study results showed microdeletions involving sY127 is significantly related to azoospermia. The high frequency of sY127 deletion 6.4% (16/250) among azoospermic patients and the significant difference between azoospermic group and oligozoospermic group in the deletion of sY127 may indicate that, microdeletions involving sY127 may associated with severe spermatogenic failure. 

As shown in table VII, although there was no significant difference between the two groups in the deletion of sequence tagged sites sY84 and sY86, deletion involving these sequences resulting in azoospermia. This is possibly attributed to the low incidence of deletion involving these sites in general, and to the small number of samples used as compared to those employed in the present study (35 oligozoospermic deletion and 101 azoospermic deletion). The present study concluded that, microdeletions involving sY127 are related to azoospermia. Deletions of sY127 as deletions of sY84, or sY86 alone may be a high risk factor of azoospermia. 
